# The Prevalence of Metabolic Syndrome Using Three Different Diagnostic Criteria among Low Earning Nomadic Kazakhs in the Far Northwest of China: New Cut-Off Points of Waist Circumference to Diagnose MetS and Its Implications

**DOI:** 10.1371/journal.pone.0148976

**Published:** 2016-02-22

**Authors:** Heng Guo, Jiaming Liu, Jingyu Zhang, Rulin Ma, Yusong Ding, Mei Zhang, Jia He, Shangzhi Xu, Shugang Li, Yizhong Yan, Lati Mu, Dongsheng Rui, Qiang Niu, Shuxia Guo

**Affiliations:** Department of Preventive Medicine, School of Medicine, Shihezi University, Shihezi, Xinjiang, 832000, China; Tulane School of Public Health and Tropical Medicine, UNITED STATES

## Abstract

**Background:**

Although the epidemic of metabolic syndrome (MetS) has aroused wide public concern, most studies on MetS tend to examine urban and high income settings, and few studies cover nomadic areas and low earning populations. This research aims to investigate the prevalence of MetS and explore the cut-off point of waist circumference in a nomadic minority typical of low income populations in the remote northwest region of China.

**Methods:**

A cross-sectional study was performed in a representative sample of 3900 Kazakh adults aged 18–84 years from 2009–2010. Three widely used criteria (ATP III\IDF\JIS) were employed to estimate the prevalence of MetS in Kazakhs to compare them with other populations. Receiver operator characteristic (ROC) curve analysis was used to explore the optimal cut-off values of waist circumference.

**Results:**

The age-adjusted prevalence of MetS was 13.8%, 20.9%, and 24.8% based on the ATP III, IDF, and JIS criteria, respectively. The prevalence of MetS was higher in women and increased with age. Except for reduced HDL-cholesterol, the risk of other components of MetS increased with waist circumference enlargement. The cut-off point of waist circumference in screening at least two other components of MetS was 88 cm in men (Sensitivity = 61.1%, Specificity = 62.1%, ROC Curve Distance = 0.54) and 83 cm in women (Sensitivity = 60.0%, Specificity = 59.6%, ROC Curve Distance = 0.57).

**Conclusion:**

The prevalence of MetS in Kazakhs is higher than the national level of China and falls in between the Euro-American and Asia levels, as their cut-off points of waist circumference differ from that recommended for Chinese. We suggest a cost-effective strategy to screen for MetS and prevent cardiovascular disease using new cut-off points of waist circumference in low earning nomadic Kazakhs.

## Introduction

Metabolic syndrome (MetS) is a reflection of the body’s metabolic disorders that cause chronic damage to organs [1–4]. It directly increases the risk of cardiovascular disease [2], type 2 diabetes mellitus and all-cause mortality [5]. The International Diabetes Federation (IDF) estimates that one-quarter of the world’s adult population has MetS [1]. MetS has become a world-wide public health issue. In China, a national survey reported that the prevalence of MetS reached 21.3% in 2008 and increased 38% compared with 13.3% in 2001[6, 7], translating to approximately 200 million adults with MetS. Because a high calorie diet and sedentary lifestyle have become increasingly popular, this figure will continue to rise. To date most investigations of MetS in China have been conducted in Han ethnic group and high-earning populations, and few studies cover the minorities and low-earning populations, especially in low-earning nomadic minorities.

This study focuses on a large nomadic minority of Kazakhs, who live in the northwest of China-Xinjiang Uyghur autonomous region (Xinjiang).Xinjiang is a multi-ethnic province with 13 main ethnic groups, among which the major ethnic groups are the Uyghur, the Han ethnic group and the Kazakh. The Kazakh is a large minority in Xinjiang with a population of 1.6 million, accounts for 7% of the total population (the Han accounts for 40% and the Uyghur accounts for 46%)[8]. The current study was performed in a typical low-income Kazakh pasturing area in the Yili prefecture located in the remote northwest region of Xinjiang, approximately 3849 kilometers from Beijing. The Yili prefecture was the largest inhabited region of Kazakhs in Xinjiang, and the participants were typical Kazakh herdsmen who live in remote mountainous pasturing region. It is difficult to perform a community health investigation in such a population due to residence dispersal. This study analyzed data from a sample size of almost 4000 Kazakh adults, making it one of the largest epidemiological surveys carried out in the Kazakh populations.

Although Kazakhs are one of five minorities with the highest risk of hypertension in China [9, 10], their health problems are seldom a concern of researchers. Characterizing the prevalence of MetS may reveal valuable information for developing appropriate policies to prevent cardiovascular disease in Kazakhs. We will study the prevalence of MetS in Kazakhs using three different criteria, including the Adult Treatment Panel III (ATP III 2001) [11], the International Diabetes Federation (IDF 2005)[12], and the Joint Interim Statement (JIS 2009)[13]. The primary difference in these criteria is the threshold value of waist circumference to diagnose abdominal obesity. Most researchers and organizations have accepted the ethnic-specific thresholds strategy [14]. Both the IDF and JIS criteria have established special cut-off points of waist circumference for the Chinese population, however, they mainly refer to the data of Chinese Hans due to lack of data in other minorities.

The Kazakhs present a typical admixture of Eastern and Western anthropometric traits[15], and they live in remote mountainous pastures for generations, with little contact with the outside world. Their special ethnicity and living environment make them different from the Hans. The mean waist circumference of Kazakhs is obviously higher than that of Hans [16]. Our research aims to identify the appropriate cut-off point of waist circumference to diagnose MetS in Kazakhs, thereby supplying valuable data for establishing an ethnic-specific threshold of waist circumference to diagnose MetS. Our observations may have important implications in public health strategies for Kazakhs and other low earning minorities in the western frontier of China.

## Materials and Methods

### Ethics statement

This study (IERB No.SHZ2010LL01) was approved by the Institutional Ethics Review Board (IERB) at the First Affiliated Hospital of Shihezi University School of Medicine. All participants were requested to sign the written informed consent before starting the investigation. Voluntary participation, anonymity and the confidentiality were guaranteed.

### Settings and participants

This survey was part of an intervention program for MetS conducted from 2009 to 2010 in the Yili prefecture of Xinjiang province. The Kazakhs are the primary minority in Yili which is approximately 3849 kilometers from Beijing. Multistage cluster random sampling was employed to choose participants. First, a representative prefecture (Yili) was identified based on the population distribution of the Kazakhs in Xinjiang. Xinyuan County was sampled randomly from Yili, and the Nalati Township was also selected randomly. The residents of 6 villages between the age 18 and 84 years were selected, and of the 3920 (1551 men and 2369 women) participants (who were interviewed to complete questionnaires, anthropometric measurements and blood tests), 3900 participants had integrated data for this current analysis.

### Anthropometric examination

Waist circumference and blood pressure were measured by trained investigators following a standard protocol [17].The waist circumference was defined as the midpoint between the lower rib and upper margin of the iliac crest at minimal respiration, as measured by a non-elastic ruler tape with an insertion buckle at one end to the nearest 0.1 cm. Blood pressure was measured using a mercury sphygmomanometer according to a standard protocol. The measurements were collected in triplicate after a 5-minute seated rest and were averaged as the blood pressure values of the individual.

### Blood plasma glucose and lipid measurements

A 5-ml blood sample after overnight fasting was collected from each participant, the samples were centrifuged at 3000 rpm for 30 min, and plasma was stored at -80°C until analysis. We tested serum glucose, HDL-cholesterol and triglycerides by a modified hexokinase enzymatic method using an Olympus AV2700 Biochemical Automatic Analyzer (Olympus, Japan) in the Biochemistry Laboratory, the First University-Affiliated Hospital of Shihezi University School of Medicine. Quality control was strictly followed regarding the procedure of blood collection, storage, and measuring processes.

### Definitions

In our research, three world-wide definitions were used to diagnose MetS to ensure comparability with those using different definitions in other studies. These definitions were from the Third Report of the National Cholesterol Education Program Expert Panel on Detection, Evaluation, and Treatment of High Blood Cholesterol in Adults in 2001 (ATPIII)[4], the International Diabetes Federation world-wide definition in 2005 (IDF) [5], and a Joint Interim Statement of multi-organizations in 2009 (JIS) [6]. The detailed information on the three criteria is listed in Table 1.

**Table 1:** Three world-wide definitions of MetS.

Risk factors	ATP criteria III (2001)	IDF criteria(2005)	JIS criteria(2009)
Abdominal obesity	Waist circumference:	Waist circumference:	Waist circumference:
(Chinese)	men≥102	men≥90	men≥85
	women≥88	women≥80	women≥80
Raised triglycerides	≥150 mg/dL (1.7 mmol/L)	≥1.7 mmol/L(150 mg/dl)	≥150 mg/dl (1.7 mmol/L)
		or treatment for this lipid	or treatment for this lipid
		abnormality	abnormality
Reduced	<40 mg/dL (1.03 mmol/L)	< 1.03 mmol/l (40 mg/dl) in	<40 mg/dl (1.0 mmol/L) in
HDL-cholesterol	in men; <50 mg/dL (1.29	men; <1.29 mmol/L (50mg	men; <50 mg/dl(1.29 mmol
	mmol/L) in women	/dl)in women; or treatment	/L)in women or treatment
		for this lipid abnormality	for this lipid abnormality
Elevated blood	Systolic≥ 130 mm Hg or/	Systolic: ≥ 130 mmHg or/	Systolic≥ 130 mmHg or/
pressure	and Diastolic≥85 mm Hg	And Diastolic: ≥ 85 mmHg	and Diastolic≥85 mmHg or
		or treatment of previously	treatment of previously
		diagnosed hypertension.	diagnosed hypertension.
Raised fasting	≥ 110 mg/dl	≥ 5.6 mmol/L (100 mg/dl)	≥ 100 mg/dl
plasma glucose		or previously diagnosed	
		type 2diabetes	

Note: MetS was diagnosed by the following: the ATP III criteria: a person had at least three of the five factors; the IDF criteria: a person had abdominal obesity plus 2 or more other risk factors; the JIS criteria: a person had at least three of the five factors.

### Statistical analysis

We established a database using EpiData software (EpiData Association, Odense, Denmark, http://www.epidata.dk/). The data were analyzed using SPSS (Statistical Program for Social Sciences, version 13.0, 2004). Continuous variables were described as the mean standard deviation (Mean ±SD) and were analyzed using the *t*-test. Categorical variables are presented as numbers and percentages and were analyzed using the Chi-square test. The official 2000 census data of China was used to calculate the age-standardized rates[18]. A two-sided statistical test was employed, and a *P* value<0.05 was considered statistically significant.

Receiver operator characteristic (ROC) curve analysis was employed to identify the optimal cut-off values of waist circumference to diagnose MetS. First, we created dichotomous variables for each cut-off value from 75 cm to 95 cm. Then, we calculated the sensitivity and specificity of each cut-off point of waist circumference for detecting at least two other components of MetS using the formulas of the diagnostic tests. Finally, we calculated the distance of the ROC curve according to the formula for the distance of the ROC curve $\left(\sqrt{{\left(1-\text{spe}cificity\right)}^{2}+{\left(1-sensitivity\right)}^{2}}\right)$ [19]. A cut-off value with the shortest distance of the ROC curve was defined as the optimal cut-off point of waist circumference to diagnose MetS.

Logistic regression was used to analyze the relationship between waist circumference and other components of MetS. Waist circumference was stratified into six levels in men and women. We calculated the odds ratios of each level compared to the lowest one adjusted for age, smoking and drinking. The trend of the odds ratios was observed to determine if there was a dose-response relationship between waist circumference and the components of MetS.

## Results

### Characteristics of the research participants

A total of 3900 participants provided information on all of the components of the MetS. There were 1,547 men (39.7%) and 2,353 women (60.3%), with an average age of (44.21±13.25) years. The mean waist circumference was (88.39±11.94) cm in men and (83.98±12.10) cm in women. The prevalence of abdominal obesity reached 60.2%, 58.6% in men and 61.3% in women, according to the JIS criteria. There was a high prevalence of hypertension (total 36.8%, 42.1% in men and 32.4% in women). Except for abdominal obesity, the most frequent individual component was elevated blood pressure in both men (57.6%) and women (45.9%), following by reduced HDL-C (44.9%) in women and raised fasting plasma glucose in men(28.1%). Additional details of these characteristics are shown in Table 2.

**Table 2:** Characteristics of the participants.

	Total	Men	Women	*t* / *χ*^*2*^	*P*
Age(years)	44.21±13.25	45.12±13.52	43.62±13.03	3.48	<0.001
Waist circumference (cm)	85.73±12.23	88.39±11.94	83.98±12.1	11.17	<0.001
BMI(kg/m^2^)	24.33±4.25	24.56±4.13	24.18±4.33	2.77	0.006
Systolic blood pressure(mmHg)	128.7±23.92	131.87±22.98	126.62±24.3	6.74	<0.001
Diastolic blood pressure(mmHg)	82.95±14.58	84.62±14.17	81.85±14.74	5.82	<0.001
Fasting Plasma glucose(mmol/L)	5.05±1.30	5.15±1.49	4.99±1.14	3.44	0.001
Serum lipids(mmol/L)					
Total triglycerides	1.26±1.01	1.39±1.17	1.16±0.87	6.68	<0.001
HDL cholesterol	1.40±0.62	1.34±0.57	1.44±0.65	5.02	<0.001
LDL cholesterol	2.40±0.87	2.46±0.91	2.36±0.84	3.55	<0.001
Total cholesterol	4.55±1.32	4.53±1.23	4.56±1.38	0.71	0.479
Abdominal obesity(cm)					
≥102/88	1074(27.5)	223(14.4)	851(36.2)	221.30	<0.001
≥90/80	2110(54.1)	667(43.1)	1443(61.3)	124.64	<0.001
≥85/80	2349(60.2)	906(58.6)	1443(61.3)	2.97	0.085
Elevated triglycerides (mmol/L)	744(19.1)	357(23.1)	387(16.4)	26.57	<0.001
Reduced HDL-cholesterol (mmol/L)	1358(34.8)	270(17.5)	1057(44.9)	313.7	<0.001
Elevated blood pressure	1970(50.5)	891(57.6)	1079(45.9)	51.45	<0.001
Raised fasting plasma glucose	961(24.6)	435(28.1)	526(22.4)	16.70	<0.001
Hypertension	1437(36.8)	652(42.1)	785(33.4)	30.95	<0.001

Note: Continuous variables were expressed by Mean ± SD and compared with *t* test. Categorical variables were expressed by n (%) and compared with *χ*^*2*^ test. Elevated blood pressure (≥130/85 mmHg), Raised fasting plasma glucose (≥5.6 mmol/L). *P* values indicate the comparison of men and women.

### Prevalence of MetS

**Fig 1:**
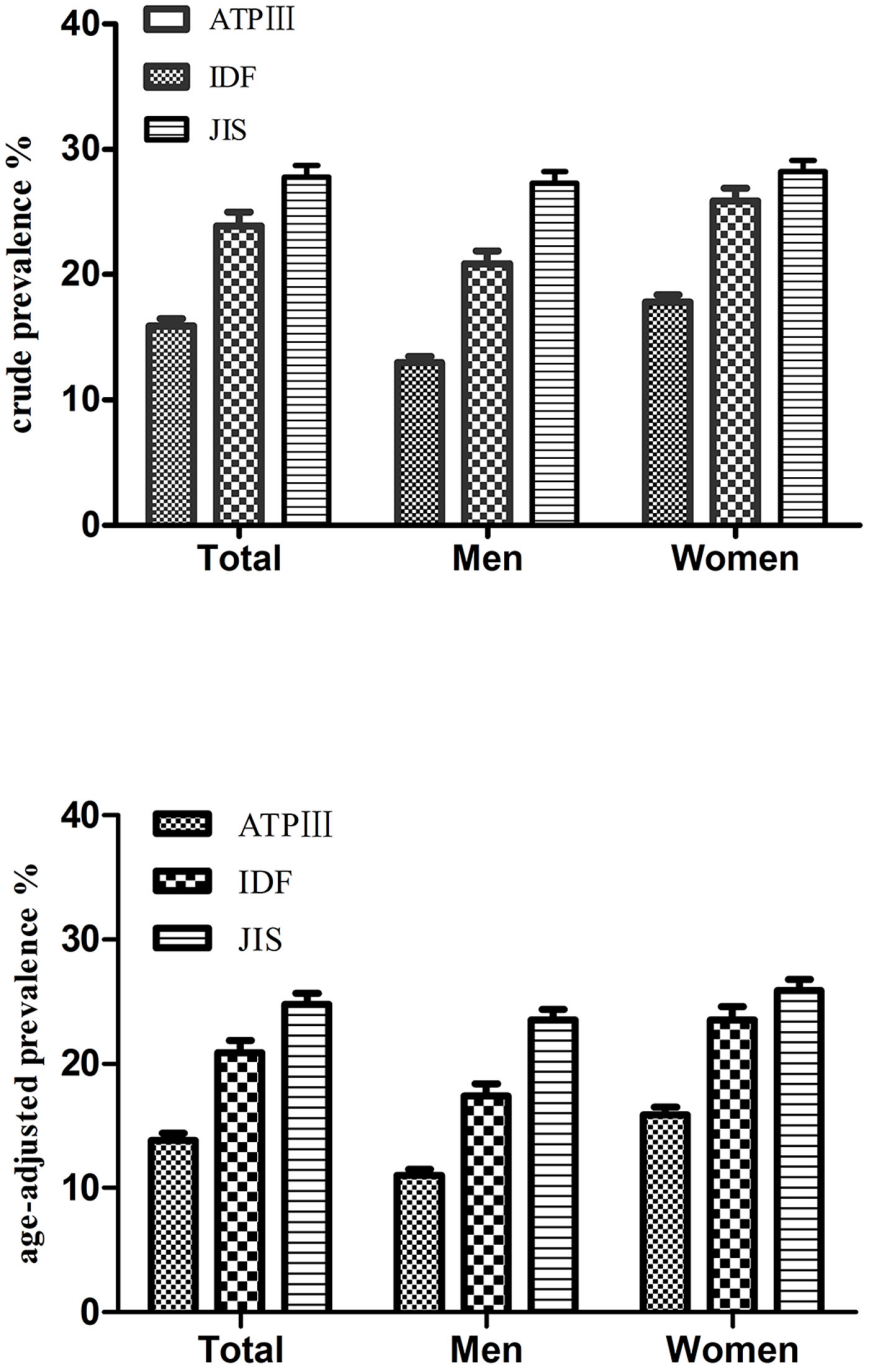
Crude and age-adjusted prevalence of MetS based on three diagnostic criteria in Kazakhs.

The overall crude prevalence of MetS was 15.9%, 23.9% and 27.8% according to the ATP III, IDF and JIS criteria, respectively. The age standardized prevalence was 13.8%, 20.9%, and 24.8%, respectively (Fig 1). Using the ATP III criteria, the prevalence in women (17.8%) was higher than in men (13.0%) (χ^2^ = 15.92, *P*<0.001); using the IDF definition, the prevalence in women (25.9%) was also higher than in men (20.9%) (χ^2^ = 12.71, *P*<0.001); however, there was no significant difference based on the JIS criteria between women (27.3%) and men (28.2%) (χ^2^ = 0.41, P = 0.521). The prevalence of MetS increased with age in both men and women according to all of the criteria and peaked at 55–64 years in men and at >65 years in women. Additional details of these prevalence rates are shown in Table 3.

**Table 3:** Age-specific and age-adjusted prevalence of MetS in Kazakh adults based on criteria of the IDF, ATP III, and JIS.

Age-group	Total (n = 3900)			Men (n = 1547)			Women (n = 2353)		
	ATPIII	IDF	JIS	ATP	IDF	JIS	ATP III	IDF	JIS
18–24	3.5 (10)	11(3.8)	25(8.7)	2(2.0)	1(1.0)	4(4.1)	8(4.2)	10(5.3)	21(11.1)
25–34	46(6.5)	81(11.5)	110(15.6)	19(6.6)	29(10.1)	49(17.1)	27(6.4)	52(12.4)	61(14.6)
35–44	113(11.4)	178(17.9)	212(21.3)	35(9.8)	57(16.0)	82(23.0)	78(12.2)	121(18.9)	130(20.3)
45–54	191(19.4)	291(29.5)	333(33.8)	59(15.4)	98(25.6)	124(32.4)	132(21.9)	193(32.1)	209(34.7)
55–64	190(28.3)	274(40.8)	302(44.9)	66(21.1)	108(34.5)	129(41.2)	124(34.5)	166(46.2)	173(48.2)
≥65	27.1 (69)	99(38.8)	104(40.8)	20(18.0)	31(27.9)	34(30.6)	49(34)	68(47.2)	70(48.6)
Total- crude	15.9 (619)	934(23.9)	1086(27.8)	201(13.0)	324(20.9)	422(27.3)	418(17.8)	610(25.9)	664(28.2)
Age- adjusted	13.8	20.9	24.8	11	17.4	23.5	15.9	23.5	25.9

Data expressed by % (n).

### Waist circumference and components of MetS

With the enlargement of waist circumference, the odds ratio of elevated blood pressure, raised fasting plasma glucose, and elevated triglycerides increased in both men and women after adjusting for age, smoking and drinking. Statistical significance was observed at almost every level of waist circumference for elevated blood pressure and elevated triglycerides. The odds ratio of reduced HDL-cholesterol was not significant at all levels of waist circumference and barely changed with waist circumference in both men and women (Table 4).

**Table 4:** Adjusted odds ratios of MetS components according to waist circumference category.

Waist (cm)	n	Elevated blood pressure		Raised plasma glucose		Elevated triglycerides		Reduced HDL-cholesterol	
		OR(95%CI)	*P*	OR(95%CI)	*P*	OR(95%CI)	*P*	OR(95%CI)	*P*
Men									
<80	367	1.00		1.00		1.00		1.00	
80–84	274	1.04 (0.75, 1.44)	0.831	1.16 (0.78, 1.70)	0.455	1.71 (1.06, 2.76)	0.028	0.81 (0.55, 1.18)	0.269
85–89	239	1.65 (1.17, 2.33)	0.004	1.09 (0.73, 1.64)	0.663	2.39 (1.49, 3.84)	<0.001	0.81 (0.54, 1.21)	0.31
90–94	242	1.85 (1.29, 2.64)	<0.001	1.40 (0.94, 2.07)	0.094	3.52 (2.22, 5.59)	<0.001	0.60 (0.39, 0.94)	0.024
95–99	142	1.63 (1.07, 2.48)	0.022	1.70 (1.09, 2.65)	0.02	5.34 (3.23, 8.82)	<0.001	0.87 (0.53, 1.41)	0.567
≥100	283	2.99 (2.07, 4.32)	<0.001	2.37 (1.64, 3.43)	<0.001	7.44 (4.78, 11.58)	<0.001	0.91 (0.61, 1.36)	0.649
Women									
<75	556	1.00		1.00		1.00		1.00	
75–79	354	1.31 (0.96, 1.77)	0.082	1.58 (1.10, 2.27)	0.013	1.33 (0.88, 2.00)	0.17	0.86 (0.65, 1.13)	0.281
80–84	403	1.35 (1.01, 1.81)	0.04	1.31 (0.91, 1.88)	0.136	1.44 (0.97, 2.14)	0.067	0.81 (0.61, 1.06)	0.13
85–89	295	1.51 (1.10, 2.08)	0.011	1.47 (1.00, 2.16)	0.047	1.85 (1.22, 2.81)	0.004	0.75 (0.55, 1.03)	0.076
90–94	275	2.03 (1.46, 2.82)	<0.001	1.54 (1.04, 2.27)	0.031	2.51 (1.66, 3.82)	<0.001	0.73(0.53, 1.01)	0.059
≥95	470	2.98 (2.20, 4.02)	<0.001	2.64 (1.88, 3.71)	<0.001	3.51 (2.41, 5.12)	<0.001	0.91 (0.68, 1.21)	0.514

Note: Adjusted for age, smoking and drinking. OR = odds ratio, CI = confidence interval.

**Fig 2:**
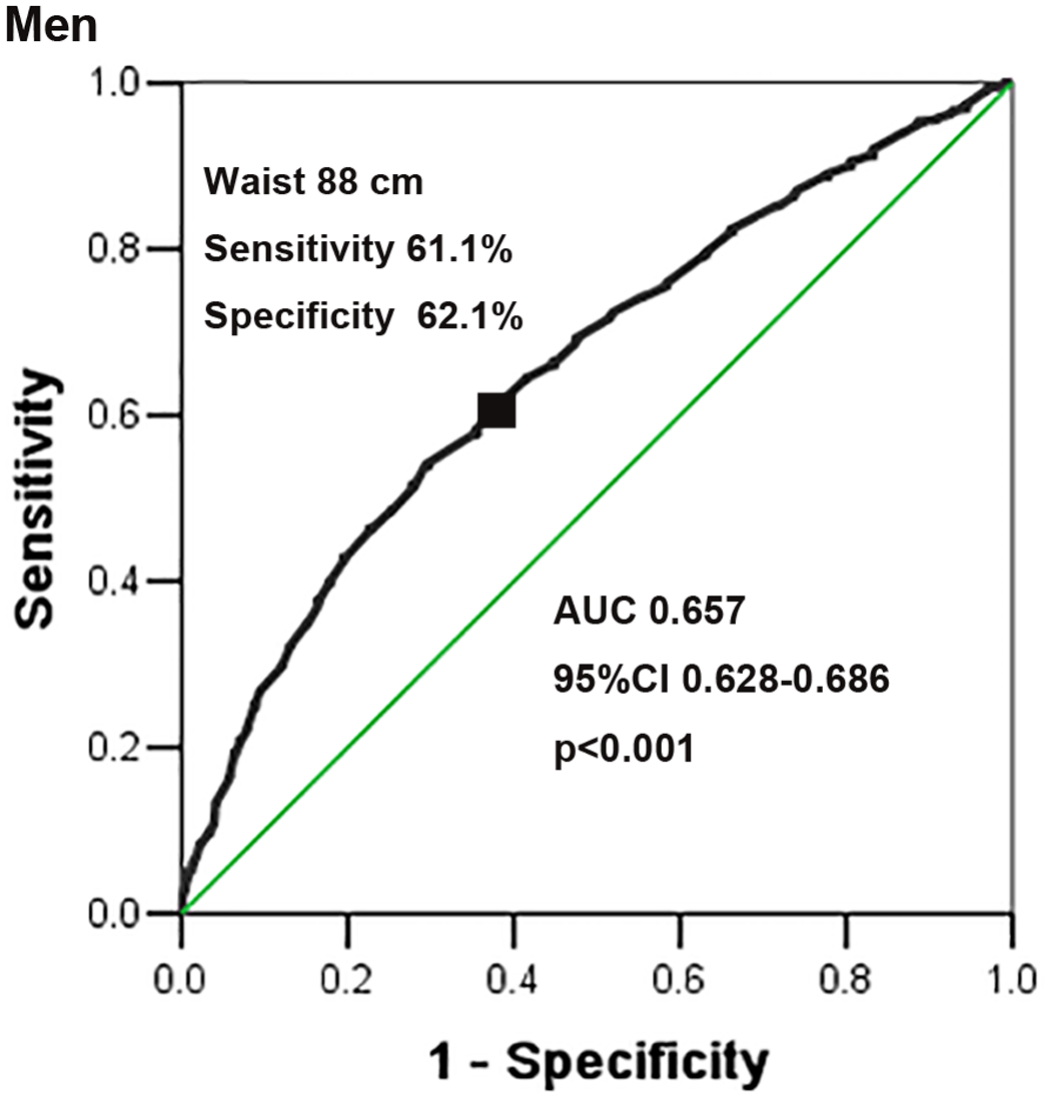
ROC curve of waist circumference to detect the existence of at least two other components of MetS in Kazakhs based on the JIS (2009) in men.

**Fig 3:**
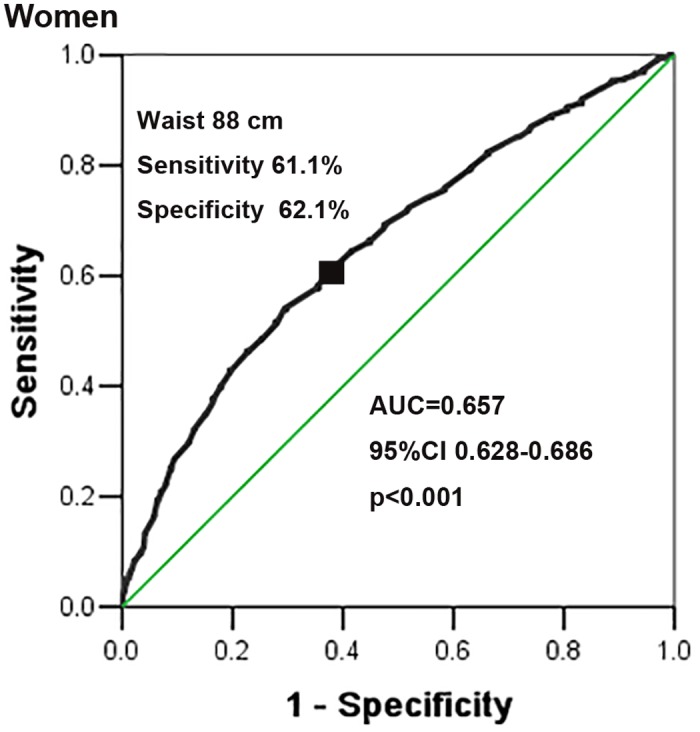
ROC curve of waist circumference to detect the existence of at least two other components of MetS in Kazakhs based on the JIS (2009) in women.

The area of the ROC curve for waist circumference for detecting the existence of at least two other components of MetS was 0.657 (95%CI 0.628, 0.686) in men (Fig 2) and 0.636 (95%CI 0.613, 0.659) in women (Fig 3). ROC curve analysis showed that the 88-cm cut-off value in men (sensitivity:61.1%, specificity:62.1%) corresponded to the shortest ROC curve distance (0.54), and 83 cm in women (sensitivity:60.0%, specificity:59.6%) corresponded to the shortest ROC curve distance (0.57). The evaluation consequences of other cut-off values are shown in Table 5.

**Table 5:** Sensitivity, specificity, and ROC curve distance for detecting clusters of at least two metabolic risk factors (based on the JIS) for each cut-off value of waist circumference.

Waist circumference (cm)		Men			Women	
	sensitivity (%)	specificity (%)	ROC curve distance	sensitivity (%)	specificity (%)	ROC curve distance
≥75	92.0	16.8	0.84	81.3	31.4	0.71
≥76	90.6	19.5	0.81	78.7	34.9	0.68
≥77	89.1	22.2	0.79	76.4	37.4	0.67
≥78	87.0	26.0	0.75	73.9	41.1	0.64
≥79	85.2	28.1	0.73	71.4	44.0	0.63
≥80	82.4	33.8	0.68	67.3	50.3	0.59
≥81	79.8	36.6	0.67	65.1	53.6	0.58
≥82	76.1	41.4	0.63	62.5	56.3	0.58
≥83^#^	74.3	44.4	0.61	60.0	59.6	0.57^#^
≥84	72.4	48.1	0.59	56.3	62.6	0.58
≥85	69.3	52.5	0.57	52.5	65.3	0.59
≥86	66.5	54.9	0.56	50.8	68.7	0.58
≥87	64.4	58.5	0.55	49.8	71.3	0.58
≥88^※^	61.1	62.1	0.54^※^	46.9	73.6	0.59
≥89	58.3	64.4	0.55	43.8	75.3	0.61
≥90	54.3	70.3	0.55	40.1	79.4	0.63
≥91	51.5	72.0	0.56	38.2	81.0	0.65
≥92	48.5	74.7	0.57	36.1	83.4	0.66
≥93	46.3	77.2	0.58	33.3	84.9	0.68
≥94	42.8	80.3	0.60	31.1	86.3	0.70
≥95	40.0	82.1	0.63	27.7	88.0	0.73

^#^ = the optimal cut-off point of waist circumference for women.

^※^ = the optimal cut-off point of waist circumference for men.

## Discussion

Although there have been many epidemiology investigations on MetS, few studies have focused on nomadic poor areas. Our survey area was located in a typical low-income remote pasturing area where approximately 95% of the population is Kazakh herdsman. Most of Kazakhs are Muslim, and they have their own language and lifestyle which are quite different from either the Hansin China or American/European populations. According to three widely used criteria, the age-standardized prevalence of MetS in the Kazakhs was 13.8% (ATPIII), 20.9% (IDF), and 24.8% (JIS). The prevalence rate was higher than that of the national level in China (18.2%,IDF)[7] and Japan(7.8%, ATPIII)[20], almost double that in a rural low-income Africa region (11.0%, IDF)[21], and lower than that in Spain (33.2%, JIS) [22] and the USA (34.3%, JIS) [23].

The Kazakhs’ dietary patterns, physical activities, medical environments, and genetics factors are strongly associated with the high prevalence of MetS. Kazakh herdsmen maintain a nomadic diet. They consume greater amounts of dairy foods (over 200 g), meat and flour, and less vegetables, beans, and even without sea foods compared with the Hans in the same region[24].They take simple food such as snacks and dairy foods as their breakfast and lunch, and they take the rich food such as meat and noodles as their dinner. They have too much high-calorie foods but they almost have no physical activities except their daily work. What’s more, they seldom receive health care due to lack of medical resources. Genetic studies revealed that Kazakhs have a mixture of Mongolian and Caucasian backgrounds[25], which may explain, at least in part, the genetic influence on the studied Kazakhs, whose prevalence for MetS is higher than that of other Asians (Han Chinese and Japanese) but lower than that of Europeans (Hispanics and American). These factors mentioned above increased the Kazakhs’ risk of developing the MetS.

Many studies have found that the prevalence of MetS increases with age without an inflection point [23, 26, 27]. Although the prevalence of MetS in Kazakhs also increases with age, it appears to decrease after 65 years old. The reason for this was that a large number of patients died before 65 due to the high mortality related with cardiovascular diseases in the Kazakhs [28, 29]. Previous studies have shown that MetS in Chinese women is more common than that in men [7, 30]. Our study also found that the prevalence in female Kazakhs was higher than in men using all of the criteria. We found an interesting trend in which the discrepancy between women and men decreased from ATP III to IDF and IDF to JIS. The biggest difference between these three criteria is the cut-off point of waist circumference to diagnose MetS. This difference indicated that waist circumference had a large influence on the prevalence of MetS. We found a dose-response relationship between waist circumference and MetS components, including elevated blood pressure, raised fasting plasma glucose, and elevated triglycerides in Kazakhs. Particularly, elevated blood pressure was intensely correlated with waist circumference. This evidence shows the benefit of screening for hypertension, which is the most common disease and the most substantial threat to Kazakhs [10, 31]. Therefore, using waist circumference as a screening tool to identify MetS in Kazakhs may prevent and help control hypertension.

In the diagnostic criteria for MetS, the cut-off point of waist circumference has been debated. A widely used criterion in ATPIII defined 102 cm in men and 88 cm in women as the cut-off points to diagnose MetS, mainly based on data from European descendants [11]. Asians have less skeletal muscle mass, low bone mineral content, and excess visceral fat for a given waist circumference [32–35]. Therefore, the IDF criterion suggests the cut-off values of waist circumference to be 90 cm in men and 80 cm in women in China [5]. However, this criterion is mainly based on the data from the southern part of China, which may not be appropriate for all Chinese. A Chinese national survey [36] including 239972 participants suggests that the appropriate cut-off values of waist circumference for Chinese adults are 85 cm in men and 80 cm in women. Another study involving 101,510 employees in Tangshan city in the central north area of China suggests that the cut-off values are 86.5 cm for men and 82.1 cm for women [37]. Nevertheless, all of the participants of these studies were mainly Hans, and few studies have explored the cut-off points of waist circumference for Chinese minorities especially the Kazakhs.

Therefore, the current study intends to supply data on the cut-off values of waist circumference to diagnose MetS in Kazakhs. We found that the average waist circumference of Kazakhs was 5 cm larger than the cut-off value of waist circumference of Hans [16]. This finding explained that the cut-off points in Kazakhs may be higher than that in Hans. In our research, ROC curve analysis indicated that 88 cm for men and 83 cm for women were the optimal cut-off points in Kazakhs, which were larger than that in Hans [36, 37]but lower than that in the Uygur minority [38]. A study of 31,076 Korean adults shows that the cut-off values detecting MetS are 83 cm in men and 76 cm in women[39]. Another Japanese study advises that the cut-off point of waist circumference for diagnosing MetS should be 85 cm in men and 78 cm in women[40].It is interesting to note that the cut-off values of Kazakhs are higher than the above reported values from the Asia population but lower than those of a European descendant population [41]. This result is consistent with the status of MetS prevalence in Kazakhs.

Most Kazakhs’ agglomerations are located in the remote mountainous area in the far northwest of China. It is difficult to seek medical advice due to the inconvenience of travel and deficiency of medical service resources. Therefore, waist circumference as a simple and cost-effective screening indicator is a perfect choice for Kazakhs to detect MetS early. Additionally, it contributes to detecting the high-risk group of hypertension cases given the significant correlation of waist circumference and hypertension in Kazakhs.

Although the participants of our study live in low-income rural communities, the prevalence of MetS is higher than the national level. Given the strong correlation between waist circumference and other components of MetS, we suggest a simple, cost-effective screening strategy for MetS in Kazakhs: measure the waist circumference and then measure the blood pressure, blood glucose, and lipid levels of individuals whose waist circumference exceeds the cut-off values (88 cm in men and 83 in women) to save medical costs and enhance the utilization efficiency of the limited health resources for Kazakh herdsmen. We recommend that this screening strategy be incorporated into routine health screening in Kazakhs, in whom a large amount of individuals with MetS could be detected, and they could be offered preventive measures before they progress to having cardiovascular diseases or type 2 diabetes. Our recommendation would not only help creating appropriate policies in preventive public health but also would enlighten other low-income minorities’ populations in China and even in the world.

A limitation of our study is its cross-sectional design. However, it is a representative and large sample of the general population of Kazakhs in China. There are few studies of Kazakhs that have explored the optimal cut-off values of waist circumference. Our observations will provide valuable data for establishing ethnic-specific thresholds of waist circumference to diagnose MetS.

## Supporting Information

S1 File Minimal data for manuscript. (SAV)

S2 File Reference data of Figs 2 and 3. (XLS)

S3 File Written permission to publish investigation pictures in PLOS ONE. (DOCX)

S4 File Editorial certificate. (PDF)

S5 File Investigation pictures. (ZIP)

S6 File Reference data of Fig 1. (DOCX)

## References

[1] Cornier MA, Dabelea D, Hernandez TL, Lindstrom RC, Steig AJ, Stob NR, et al The metabolic syndrome. Endocr Rev. 2008;29(7):777–822. 10.1210/er.2008-0024 . 18971485 PMC5393149

[2] Mottillo S, Filion KB, Genest J, Joseph L, Pilote L, Poirier P, et al The metabolic syndrome and cardiovascular risk a systematic review and meta-analysis. J Am Coll Cardiol. 2010;56(14):1113–32. 10.1016/j.jacc.2010.05.034 . 20863953

[3] Kaya E, Sikka SC, Gur S. A Comprehensive Review of Metabolic Syndrome Affecting Erectile Dysfunction. J Sex Med. 2015 10.1111/jsm.12828 . 25675988

[4] Alberti KG, Zimmet P, Shaw J, Group IDFETFC. The metabolic syndrome-a new worldwide definition. Lancet. 2005;366(9491):1059–62. 10.1016/S0140-6736(05)67402-8 . 16182882

[5] Saito I. Epidemiological evidence of type 2 diabetes mellitus, metabolic syndrome, and cardiovascular disease in Japan. Circulation journal: official journal of the Japanese Circulation Society. 2012;76(5):1066–73. . 22453006 10.1253/circj.cj-11-1519

[6] Further Study of Risk Factors for Stroke Coronary Heart Disease Group. The prevalence of metabolic syndrome in a 11 provinces cohort in China. Zhonghua Yu Fang Yi Xue Za Zhi. 2002;36(5):298–300. . 12411186

[7] Xi B, He D, Hu Y, Zhou D. Prevalence of metabolic syndrome and its influencing factors among the Chinese adults: the China Health and Nutrition Survey in 2009. Prev Med. 2013;57(6):867–71. 10.1016/j.ypmed.2013.09.023 24103567 PMC4044099

[8] Statistic Bureau of Xinjiang Uyghur Autonomous Region. Xinjiang Statistical Yearbook 2014. Available: http://www.xjtj.gov.cn/sjcx/tjnj_3415/2014xjtjnj/rkjy_2014/201506/t20150630_471951.html. Accessed 24 Dec 2015.

[9] Yan WL, Li XS, Wang Q, Huang YD, Zhang WG, Zhai XH, et al Overweight, high blood pressure and impaired fasting glucose in Uyghur, Han, and Kazakh Chinese children and adolescents. Ethn Health. 2015;20(4):365–75. 10.1080/13557858.2014.921894 24904957 PMC4258184

[10] Liu F, Ma YT, Yang YN, Zhen YJ, Xie X, Li XM, et al The prevalence of isolated systolic hypertension in adult populations from the Han, Uygur and Kazakh ethnic groups in Xinjiang, China. Blood Press. 2014;23(3):154–9. 10.3109/08037051.2013.838827 . 24070221

[11] Expert Panel on Detection E, Treatment of High Blood Cholesterol in A. Executive Summary of The Third Report of The National Cholesterol Education Program (NCEP) Expert Panel on Detection, Evaluation, And Treatment of High Blood Cholesterol In Adults (Adult Treatment Panel III). JAMA. 2001;285(19):2486–97. . 11368702 10.1001/jama.285.19.2486

[12] Alberti KG, Zimmet P, Shaw J. Metabolic syndrome—a new world-wide definition. A Consensus Statement from the International Diabetes Federation. Diabet Med. 2006;23(5):469–80. 10.1111/j.1464-5491.2006.01858.x . 16681555

[13] Alberti KG, Eckel RH, Grundy SM, Zimmet PZ, Cleeman JI, Donato KA, et al Harmonizing the metabolic syndrome: a joint interim statement of the International Diabetes Federation Task Force on Epidemiology and Prevention; National Heart, Lung, and Blood Institute; American Heart Association; World Heart Federation; International Atherosclerosis Society; and International Association for the Study of Obesity. Circulation. 2009;120(16):1640–5. 10.1161/CIRCULATIONAHA.109.192644 . 19805654

[14] Misra A, Wasir JS, Vikram NK. Waist circumference criteria for the diagnosis of abdominal obesity are not applicable uniformly to all populations and ethnic groups. Nutrition. 2005;21(9):969–76. 10.1016/j.nut.2005.01.007 . 15993041

[15] Black ML, Wise CA, Wang W, Bittles AH. Combining genetics and population history in the study of ethnic diversity in the People's Republic of China. Hum Biol. 2006;78(3):277–93. 10.1353/hub.2006.0041 . 17216801

[16] Du H, Bennett D, Li L, Whitlock G, Guo Y, Collins R, et al Physical activity and sedentary leisure time and their associations with BMI, waist circumference, and percentage body fat in 0.5 million adults: the China Kadoorie Biobank study. Am J Clin Nutr. 2013;97(3):487–96. 10.3945/ajcn.112.046854 23364014 PMC4345799

[17] Chobanian AV, Bakris GL, Black HR, Cushman WC, Green LA, Izzo JL Jr., et al The Seventh Report of the Joint National Committee on Prevention, Detection, Evaluation, and Treatment of High Blood Pressure: the JNC 7 report. JAMA. 2003;289(19):2560–72. 10.1001/jama.289.19.2560 . 12748199

[18] National Bureau of Statistics of China. Tabulation on the 2000 population census of People’s Republic of China. Available: http://www.stats.gov.cn/tjsj/pcsj/rkpc/5rp/index.htm. Accessed 20 Mar 2015.

[19] Zhou BF, Wu YF, Li Y, Zhang LF. [The cut-off point of waist circumference for identifying metabolic syndrome in Chinese adults]. Zhonghua Xin Xue Guan Bing Za Zhi. 2005;33(1):81–5. . 15924790

[20] Arai H, Yamamoto A, Matsuzawa Y, Saito Y, Yamada N, Oikawa S, et al Prevalence of metabolic syndrome in the general Japanese population in 2000. J Atheroscler Thromb. 2006;13(4):202–8. . 16908953 10.5551/jat.13.202

[21] Ntandou G, Delisle H, Agueh V, Fayomi B. Abdominal obesity explains the positive rural-urban gradient in the prevalence of the metabolic syndrome in Benin, West Africa. Nutr Res. 2009;29(3):180–9. 10.1016/j.nutres.2009.02.001 . 19358932

[22] Marcuello C, Calle-Pascual AL, Fuentes M, Runkle I, Rubio MA, Montanez C, et al Prevalence of the metabolic syndrome in Spain using regional cutoff points for waist circumference: the diabetes study. Acta Diabetol. 2013;50(4):615–23. Epub 2013/03/21. 10.1007/s00592-013-0468-8 . 23512475

[23] Ford ES, Li C, Zhao G. Prevalence and correlates of metabolic syndrome based on a harmonious definition among adults in the US. J Diabetes. 2010;2(3):180–93. 10.1111/j.1753-0407.2010.00078.x . 20923483

[24] Zhai F, He Y, Wang Z, Hu Y. Status and characteristic of dietary intake of 12 minority nationalities in China. Wei Sheng Yan Jiu. 2007;36(5):539–41. . 18095560

[25] Wang W, Wise C, Baric T, Black ML, Bittles AH. The origins and genetic structure of three co-resident Chinese Muslim populations: the Salar, Bo'an and Dongxiang. Hum Genet. 2003;113(3):244–52. 10.1007/s00439-003-0948-y . 12759817

[26] Gu D, Reynolds K, Wu X, Chen J, Duan X, Reynolds RF, et al Prevalence of the metabolic syndrome and overweight among adults in China. Lancet. 2005;365(9468):1398–405. 10.1016/S0140-6736(05)66375-1 . 15836888

[27] Ford ES, Giles WH, Dietz WH. Prevalence of the metabolic syndrome among US adults: findings from the third National Health and Nutrition Examination Survey. JAMA. 2002;287(3):356–9. . 11790215 10.1001/jama.287.3.356

[28] Jiang J, Zhang B, Zhang M, Xue F, Tang Y, Liang S, et al Prevalence of conventional cardiovascular disease risk factors among Chinese Kazakh individuals of diverse occupational backgrounds in Xinjiang China. Int J Cardiol. 2015;179:558–60. 10.1016/j.ijcard.2014.10.077 . 25466562

[29] Ma X, Bai X, Karmacharya UK, Huang D, Huang Y, Xie X, et al Cardiovascular risks in Kazakh population in Xinjiang Province of China. Ethn Dis. 2014;24(3):316–20. . 25065073

[30] Song QB, Zhao Y, Liu YQ, Zhang J, Xin SJ, Dong GH. Sex difference in the prevalence of metabolic syndrome and cardiovascular-related risk factors in urban adults from 33 communities of China: The CHPSNE study. Diab Vasc Dis Res. 2015 10.1177/1479164114562410 . 25670848

[31] Lu Z, Lu Z, Zhu Y, Yan Z, Liu X, Yan W, et al Enhanced hypertension prevalence in non-Han Chinese minorities from Xinjiang Province, China. Hypertens Res. 2009;32(12):1097–103. 10.1038/hr.2009.159 19779488 PMC2854648

[32] Banerji MA, Faridi N, Atluri R, Chaiken RL, Lebovitz HE. Body composition, visceral fat, leptin, and insulin resistance in Asian Indian men. J Clin Endocrinol Metab. 1999;84(1):137–44. 10.1210/jcem.84.1.5371 . 9920074

[33] Song MY, Kim J, Horlick M, Wang J, Pierson RN Jr., Heo M, et al Prepubertal Asians have less limb skeletal muscle. J Appl Physiol (1985). 2002;92(6):2285–91. 10.1152/japplphysiol.01066.2001 . 12015338

[34] Misra A, Vikram NK. Insulin resistance syndrome (metabolic syndrome) and obesity in Asian Indians: evidence and implications. Nutrition. 2004;20(5):482–91. 10.1016/j.nut.2004.01.020 . 15105039

[35] Wagner DR, Heyward VH. Measures of body composition in blacks and whites: a comparative review. Am J Clin Nutr. 2000;71(6):1392–402. . 10837277 10.1093/ajcn/71.6.1392

[36] Zhou BF, Cooperative Meta-Analysis Group of the Working Group on Obesity in C. Predictive values of body mass index and waist circumference for risk factors of certain related diseases in Chinese adults-study on optimal cut-off points of body mass index and waist circumference in Chinese adults. Biomed Environ Sci. 2002;15(1):83–96. . 12046553

[37] Wang F, Wu S, Song Y, Tang X, Marshall R, Liang M, et al Waist circumference, body mass index and waist to hip ratio for prediction of the metabolic syndrome in Chinese. Nutr Metab Cardiovasc Dis. 2009;19(8):542–7. 10.1016/j.numecd.2008.11.006 . 19188050

[38] Lu Q, Xie Z, Zhang H, Ren J. Evaluation of the appropriate diagnostic threshold of waist circumference for the cardiometabolic syndrome in Chinese Uygur adults. J Cardiometab Syndr. 2009;4(2):120–5. 10.1111/j.1559-4572.2008.00045.x . 19614800

[39] Kim HK, Kim CH, Park JY, Lee KU. Lower waist-circumference cutoff point for the assessment of cardiometabolic risk in Koreans. Diabetes Res Clin Pract. 2009;85(1):35–9. 10.1016/j.diabres.2009.04.009 . 19410320

[40] Hara K, Matsushita Y, Horikoshi M, Yoshiike N, Yokoyama T, Tanaka H, et al A proposal for the cutoff point of waist circumference for the diagnosis of metabolic syndrome in the Japanese population. Diabetes Care. 2006;29(5):1123–4. Epub 2006/04/29. 10.2337/diacare.2951123 . 16644651

[41] Molarius A, Seidell JC, Sans S, Tuomilehto J, Kuulasmaa K. Varying sensitivity of waist action levels to identify subjects with overweight or obesity in 19 populations of the WHO MONICA Project. J Clin Epidemiol. 1999;52(12):1213–24. . 10580785 10.1016/s0895-4356(99)00114-6

