# Supplementary figures and images for: The Prevalence of Metabolic Syndrome Using Three Different Diagnostic Criteria among Low Earning Nomadic Kazakhs in the Far Northwest of China: New Cut-Off Points of Waist Circumference to Diagnose MetS and Its Implications
Source: PLoS One. 2016 Feb 22;11(2):e0148976. doi: 10.1371/journal.pone.0148976 (PMC4763161; doi:10.1371/journal.pone.0148976)

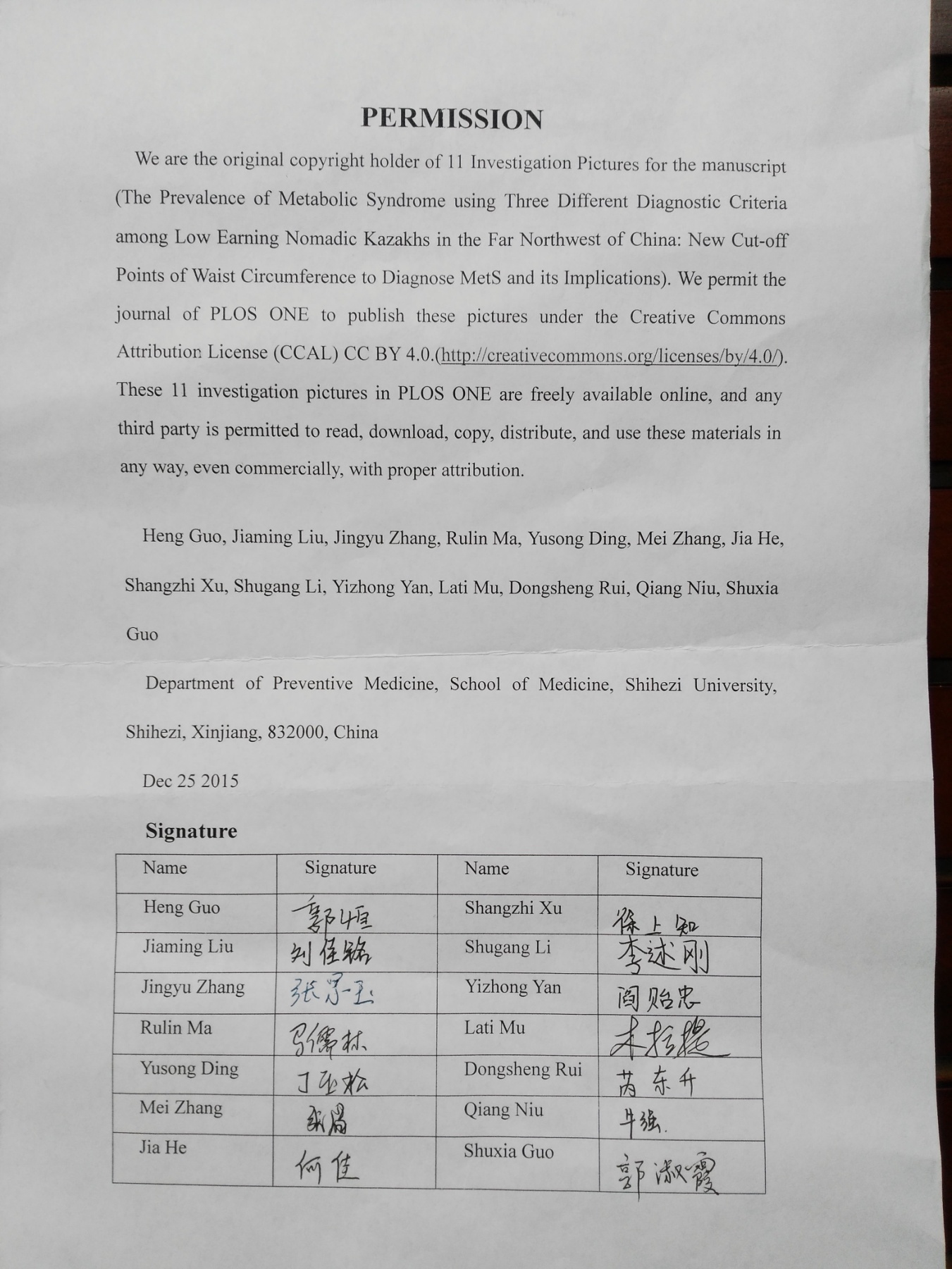

Supplement: S3 File — (DOCX) [file pone.0148976.s003.docx]

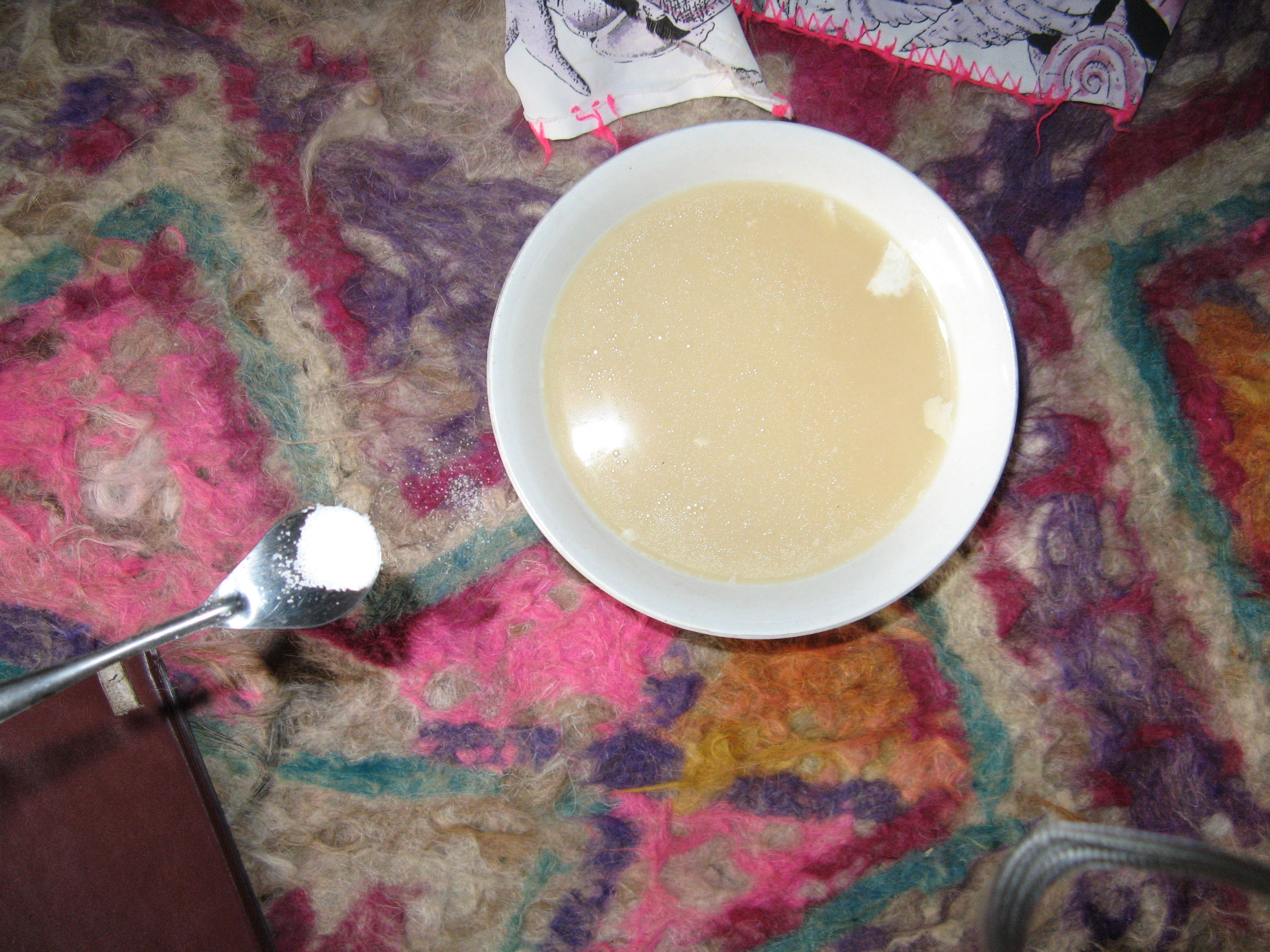

Supplement: S5 File — (ZIP) [file pone.0148976.s005.zip › investigation pictures-NEW (delete identifiable images of people)/IMG_0739.JPG]

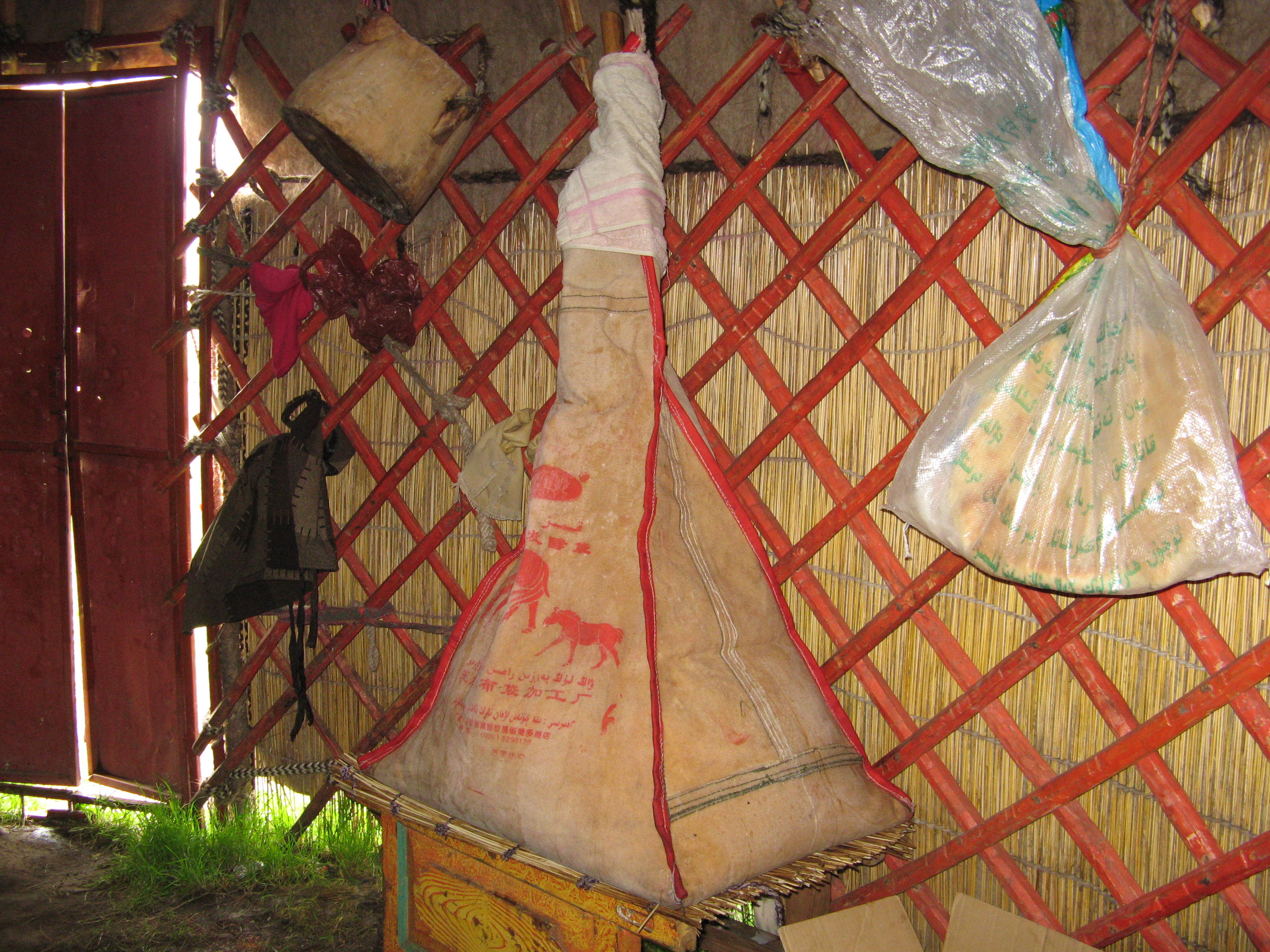

Supplement: S5 File — (ZIP) [file pone.0148976.s005.zip › investigation pictures-NEW (delete identifiable images of people)/IMG_1959.JPG]

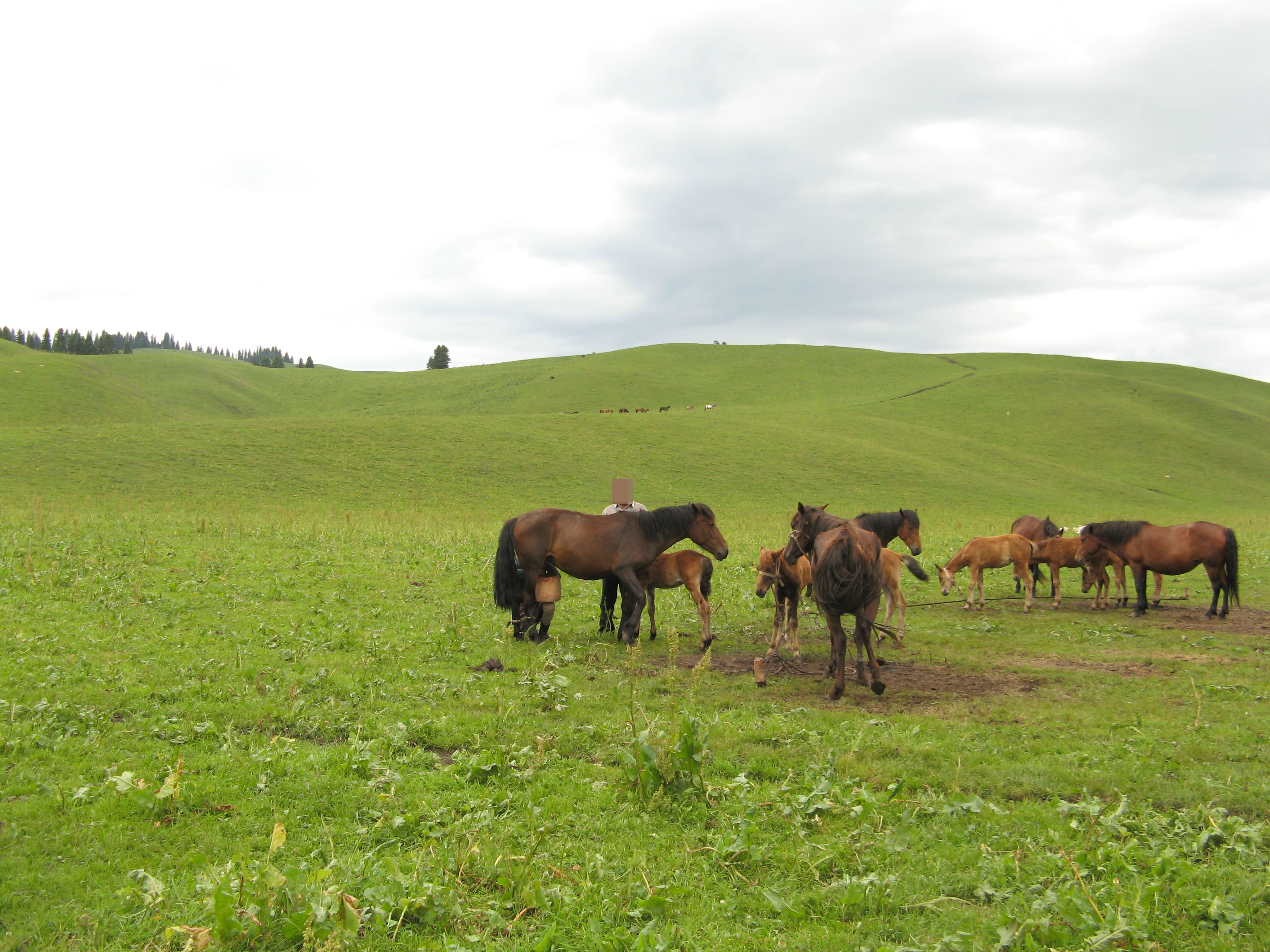

Supplement: S5 File — (ZIP) [file pone.0148976.s005.zip › investigation pictures-NEW (delete identifiable images of people)/IMG_1972.JPG]

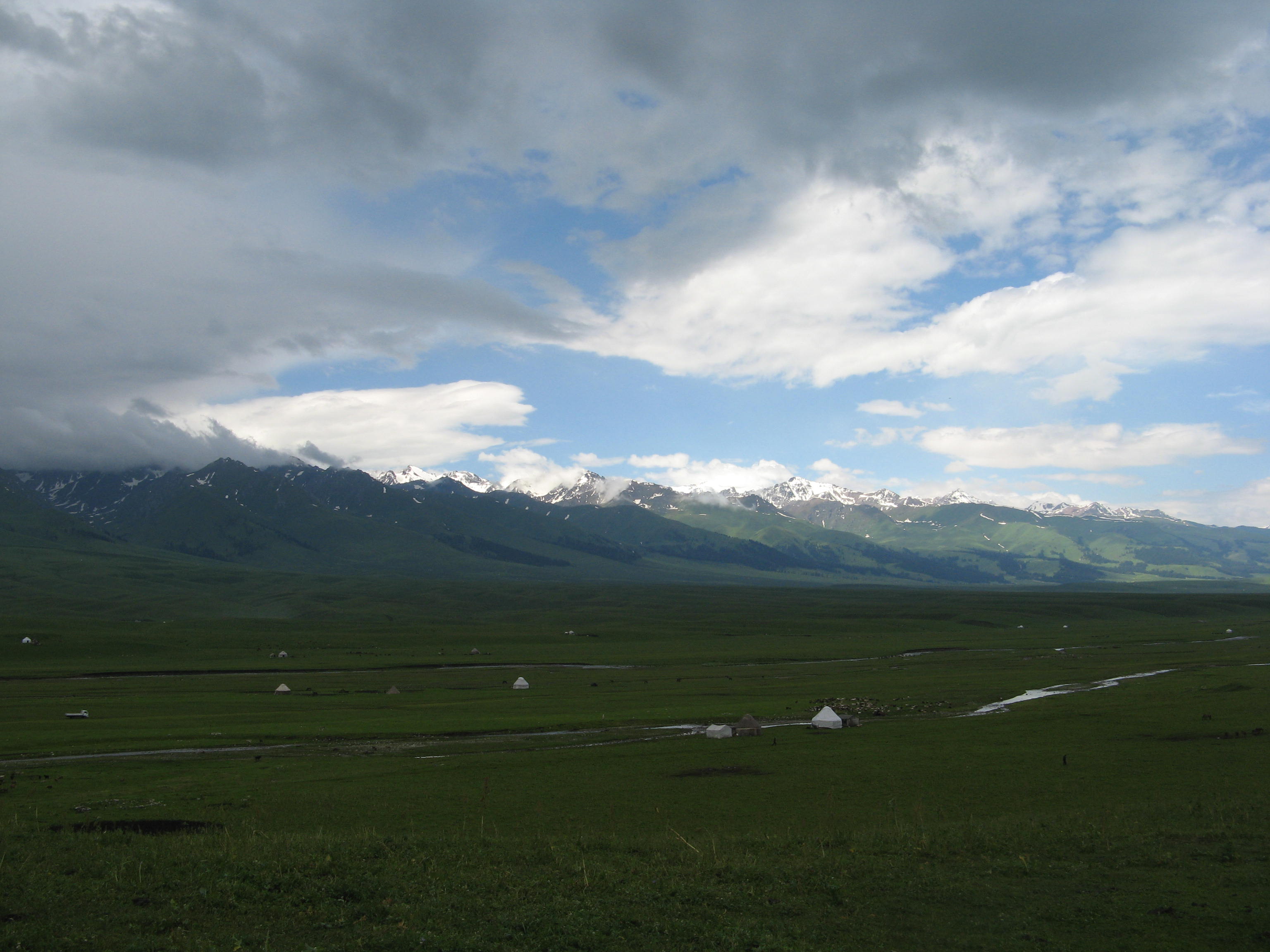

Supplement: S5 File — (ZIP) [file pone.0148976.s005.zip › investigation pictures-NEW (delete identifiable images of people)/IMG_1988.JPG]
