# Supplementary material for: The Prevalence of Metabolic Syndrome Using Three Different Diagnostic Criteria among Low Earning Nomadic Kazakhs in the Far Northwest of China: New Cut-Off Points of Waist Circumference to Diagnose MetS and Its Implications
Source: PLoS One. 2016 Feb 22;11(2):e0148976. doi: 10.1371/journal.pone.0148976 (PMC4763161; doi:10.1371/journal.pone.0148976)
Supplement: S4 File — (PDF) [file pone.0148976.s004.pdf]

AJE AMERICAN JOURNAL EXPERTS

EDITORIAL CERTIFICATE

This document certifies that the manuscript listed below was edited for proper English language, grammar, punctuation, spelling, and overall style by one or more of the highly qualified native English speaking editors at American Journal Experts.

Manuscript title:

The Prevalence of Metabolic Syndrome in Three Different Diagnostic Criteria among low Earning Nomadic Kazakhs in Far Northwest of China: A New Cut-off Points of Waist Circumference to Diagnose Metabolic Syndrome and Its Implication

Authors:

Heng Guo, Jiaming Liu, Jingyu Zhang, Rulin Ma, Yusong Ding, Mei Zhang, Jia He, Shangzhi Xu, Shugang Li, Yizhong Yan, Lati Mu, Dongsheng Rui, Qiang Niu, Shuxia Guo*

Date Issued:

August 21, 2015

Certificate Verification Key:

C436-0697-5428-4517-8697

This certificate may be verified at www.aje.com/certificate. This document certifies that the manuscript listed above was edited for proper English language, grammar, punctuation, spelling, and overall style by one or more of the highly qualified native English speaking editors at American Journal Experts. Neither the research content nor the authors' intentions were altered in any way during the editing process. Documents receiving this certification should be English-ready for publication; however, the author has the ability to accept or reject our suggestions and changes. To verify the final AJE edited version, please visit our verification page. If you have any questions or concerns about this edited document, please contact American Journal Experts at support@aje.com.

American Journal Experts provides a range of editing, translation and manuscript services for researchers and publishers around the world. Our top-quality PhD editors are all native English speakers from America's top universities. Our editors come from nearly every research field and possess the highest qualifications to edit research manuscripts written by non-native English speakers. For more information about our company, services and partner discounts, please visit www.aje.com.
