# Supplementary material for: The Prevalence of Metabolic Syndrome Using Three Different Diagnostic Criteria among Low Earning Nomadic Kazakhs in the Far Northwest of China: New Cut-Off Points of Waist Circumference to Diagnose MetS and Its Implications
Source: PLoS One. 2016 Feb 22;11(2):e0148976. doi: 10.1371/journal.pone.0148976 (PMC4763161; doi:10.1371/journal.pone.0148976)
Supplement: S6 File — (DOCX) [file pone.0148976.s006.docx]

**Table 1 Crude and age-adjusted prevalence of MetS based on three diagnostic criteria in Kazakhs**

| Age-group | Total (n=3900) | | |  | Men (n=1547) | | |  | Women (n=2353) | | |
| --- | --- | --- | --- | --- | --- | --- | --- | --- | --- | --- | --- |
|  | ATPⅢ | IDF | JIS |  | ATP | IDF | JIS |  | ATP Ⅲ | IDF | JIS |
| Total- crude | 15.9 (619) | 934(23.9) | 1086(27.8) |  | 201(13.0) | 324(20.9) | 422(27.3) |  | 418(17.8) | 610(25.9) | 664(28.2) |
| Age- adjusted | 13.8 | 20.9 | 24.8 |  | 11 | 17.4 | 23.5 |  | 15.9 | 23.5 | 25.9 |
